# Gram‐negative microbiota is related to acute exacerbation in children with asthma

**DOI:** 10.1002/clt2.12069

**Published:** 2021-10-12

**Authors:** Yoon Hee Kim, Haerin Jang, Soo Yeon Kim, Jae Hwa Jung, Ga Eun Kim, Mi Reu Park, Jung Yeon Hong, Mi Na Kim, Eun Gyul Kim, Min Jung Kim, Kyung Won Kim, Myung Hyun Sohn

**Affiliations:** ^1^ Department of Pediatrics Gangnam Severance Hospital Seoul Korea; ^2^ Institute of Allergy Severance Biomedical Science Institute Brain Korea 21 Project for Medical Science Yonsei University College of Medicine Seoul Korea; ^3^ Department of Pediatrics Severance Hospital Seoul Korea; ^4^ Division of Cardiovascular Disease Research Department for Chronic Disease Convergence Research Korea National Institute of Health Cheongju Korea; ^5^ Department of Pediatrics Yongin Severance Hospital Yongin Korea

**Keywords:** asthma, children, induced sputum, lipopolysaccharide, microbiome, asthma, induzierter auswurf, kinder, lipopolysaccharid, mikrobiom

## Abstract

**Background:**

The upper‐airway microbiota may be associated with the pathogenesis of asthma and useful for predicting acute exacerbation. However, the relationship between the lower‐airway microbiota and acute exacerbation in children with asthma is not well understood. We evaluated the characteristics of the airway microbiome using induced sputum from children with asthma exacerbation and compared the microbiota‐related differences of inflammatory cytokines with those in children with asthma.

**Methods:**

We analysed the microbiome using induced sputum during acute exacerbation of asthma in children. We identified microbial candidates that were prominent in children with asthma exacerbation and compared them with those in children with stable asthma using various analytical methods. The microbial candidates were analysed to determine their association with inflammatory cytokines. We also developed a predictive functional profile using PICRUSt.

**Results:**

A total of 95 children with allergic sensitisation including 22 with asthma exacerbation, 67 with stable asthma, and 6 controls were evaluated. We selected 26 microbial candidates whose abundances were significantly increased, decreased, or correlated during acute exacerbation in children with asthma. Among the microbial candidates, *Campylobacter*, *Capnocytophaga*, *Haemophilus*, and *Porphyromonas* were associated with inflammatory cytokines including macrophage inflammatory protein (MIP)‐1β, programmed death‐ligand 1, and granzyme B. Both *Campylobacter* and MIP‐1β levels were correlated with sputum eosinophils. Increased lipopolysaccharide biosynthesis and decreased glycan degradation were observed in children with asthma exacerbation.

**Conclusion:**

Gram‐negative microbes in the lower airway were related to acute exacerbation in children with asthma. These microbes and associated cytokines may play a role in exacerbating asthma in children.

## 1 INTRODUCTION

Acute exacerbation of asthma in children is remains difficult to treat and can result in severe morbidity including deteriorated lung function and mortality.^1^ Viral respiratory infections are considered as main triggering factor in children with asthma exacerbation and may be influenced by risk factors such as allergen sensitisation and exposure, antiviral immunity, and genetic predisposition.^2^, ^3^ Numerous studies of the airway microbiome using culture‐independent next‐generation sequencing methods for isolating microbes have suggested that host factors are related to asthma exacerbation.^4^

Although which respiratory sample is the most appropriate for microbiome analysis is currently unclear, nasal samples are generally evaluated in children with asthma to predict acute exacerbation because they are easy to collect, particularly from children. Many studies of the respiratory microbiome have indicated that using nasal samples is useful for predicting biomarkers in children with asthma exacerbation.^5^, ^6^ However, nasal samples mainly reflect the upper airway, and induced sputum samples may be superior to nasal or oral samples for assessing the bronchial microbiota composition.^7^ As asthma exacerbation may develop from an exaggerated lower airway response to an environmental stimulus and acute severe lower airway inflammation, respiratory microbiome analysis using induced sputum could be useful for understanding the pathophysiology of asthma exacerbation.^8^

In this study, we evaluated the characteristics of the airway microbiome using induced sputum in children with asthma exacerbation. In addition, by comparing inflammatory cytokines with distinct microbiota in acute exacerbation, we predicted the role of the airway microbiota in children with asthma exacerbation.

## 2 METHODS

### 2.1 Subjects

We enrolled children who visited the Severance Children's Hospital for work‐up or treatment of asthma or routine health check‐up from December 2012 to September 2018.

We defined three groups of patients: asthma exacerbation, stable asthma, and respiratory heathy control groups. Asthma was diagnosed based on current episodic respiratory symptoms such as recurrent cough or dyspnoea, shortness of breath, chest tightness, and airway hyperresponsiveness or bronchodilator response according to the guidelines of the American Thoracic Society.^9^ Stable asthma was defined as no asthmatic exacerbation during the preceding 4 weeks accompanied by the need for systemic corticosteroids or an increased use of inhaled corticosteroids, the use of rescue treatment ≤3 times per week, and no clinical indication for a change in medication. Asthma exacerbation was defined as the worsening of asthma requiring the use of systemic corticosteroids or hospitalisation to prevent a serious outcome.^10^ Respiratory healthy controls had normal lung function without airway hyperresponsiveness and had never had a doctor's diagnosis of asthma.

The stable asthma and healthy control groups were enrolled in an outpatient clinic and underwent spirometry, sputum induction, and blood sampling at the first visit followed by provocholine challenge test at the second visit. The asthma exacerbation group was admitted to the hospital because of worsening of asthma symptoms and underwent spirometry, sputum induction, blood sampling, and nasopharyngeal swab within 24 h after hospitalisation. The nasopharyngeal swabbed samples were analysed for 12 common respiratory viruses with a multiplex PCR/RT‐PCT kit (SolGent, Daejeon, Korea).^11^ These viruses included human rhinovirus, respiratory syncytial virus, human bocavirus, influenza A and B virus, human metapneumovirus, adenovirus, human coronaviruses 229E, OC43, and parainfluenza viruses 1–3.

In the blood samples, total serum IgE and specific IgE levels were measured by the Pharmacia CAP assay (Uppsala, Sweden). A specific IgE test was performed for common allergens in Korea, including 2 types of dust mites such as *Dermatophagoides pteronyssinus* and *Dermatophagoides farina*, cat and dog epithelium, and cockroach, as well as mould and pollen allergens, including Alternaria, birch, mugwort, Japanese hop, and ragweed. Atopy was defined as ≥0.35 KUa/L specific IgE to more than one allergen.

Sputum induction and processing methods are described in detail in the Supplementary Methods. For the fidelity of the induced sputum collected, the proportion of squamous cells was checked and the samples were considered acceptable for analysis when the squamous cell proportion was <20% of the total cells.^12^ For microbiome analysis, the DNA extraction, PCR amplification and sequencing, and bioinformatics analysis procedures are also described in the Supplementary Methods.

This study was approved by the Institutional Review Board of Severance Hospital (protocol no. 4‐2004‐0036). Written informed consent was obtained from the participants and their parents.

### 2.2 Evaluating the microbial candidates as biomarkers of asthma exacerbation

For biomarker discovery in children with asthma exacerbation, we used three different types of data analysis methods including the linear discriminant analysis effect size (LEfSe) method,^13^ similarity of percentages (SIMPER) analysis,^14^ and microbiota network analysis using SparCC.^15^ In using LEfSe analysis, we defined the microbial candidates as those showing significant LEfSe values in children with asthma exacerbation compared to those in both children with stable asthma and controls. SIMPER analysis was performed to define the microbial candidates that could explain the difference in microbiome composition between children with asthma exacerbation and those with stable asthma with up to 80% dissimilarity. Microbiota network analysis was performed using a correlation coefficient of *r* > 0.25 or *r* < −0.25 and *p* value < 0.01, and the results were visualised with Cytoscape (version 3.4.0) software for children with asthma exacerbation. We identified the microbial candidates showing significant correlations with each other.

Based on the functional profiles predicted by the PICRUSt^16^ and MinPath algorithms,^17^ functional biomarkers were identified by LEfSe analysis among the three groups. All analytical methods were performed in EzBioCloud 16S‐based MTP, the ChunLab's bioinformatics cloud platform.

### 2.3 Cytokine analysis

Cytokine analysis of sputum was performed in children with asthma exacerbation and stable asthma. A human fixed immunotherapy discovery magnetic panel‐24 plex kit (Magnetic Luminex® Performance Assay multiplex kit, R&D Systems, Minneapolis, MN, USA) was used for cytokine analysis. This kit was used to analyse the cluster of differentiation 40, granulocyte–macrophage colony‐stimulating factor, granzyme B, interferon‐α, interferon‐γ, interleukin (IL)‐1α, IL‐1β, IL‐1Ra, IL‐2, IL‐4, IL‐6, IL‐8, IL‐10, IL‐12p70, IL‐13, IL‐15, IL‐17A, IL‐33, C‐X‐C motif chemokine 10, monocyte chemoattractant protein‐1, macrophage inflammatory proteins (MIP)‐1α, MIP‐1β, programmed death‐ligand (PD‐L) 1, and tumour necrosis factor‐α.

### 2.4 Statistical analysis

Subjects' characteristics were compared among children with asthma exacerbation, those with stable asthma, and controls using Kruskal–Wallis test and pairwise comparison. To assess the relationship between the microbial candidates and the significant inflammatory cytokines in children with asthma exacerbation, Spearman's rank correlation was used. Correction for multiple comparisons was carried out using the Benjamini–Hochberg false discovery rate method. To display the results graphically, we plotted a correlation matrix showing the relationships between the microbial candidates and the significant inflammatory cytokines in children with asthma exacerbation. All *p*‐values < 0.05 were considered as statistically significant. SPSS version 23 statistical software (SPSS, Inc., Chicago, IL, USA) and R statistical package (R version 3.2.5.; Institute for Statistics and Mathematics, Vienna, Austria; www.R‐project.org) were used.

## 3 RESULTS

### 3.1 Clinical characteristics

A total of 127 children including 22 children with asthma exacerbation, 83 with stable asthma, and 22 controls were enrolled. All children with asthma exacerbation showed atopy. Because atopy in asthma has been suggested to strongly influence the human microbiome,^18^, ^19^ we excluded children without atopy. A total of 95 children with allergic sensitisation including 22 with asthma exacerbation, 67 with stable asthma, and 6 controls were evaluated.

The subjects’ clinical characteristics are shown in Table 1. The median ages of children with asthma exacerbation and stable asthma were 9.0 and 8.0 years, percentages of males were 68.2% and 74.6%, and median total IgE levels were 484 and 439 IU/ml, respectively. In children with asthma exacerbation, the blood and sputum eosinophil were 460/mm^2^ and 3.0%. Among the 22 children with asthma exacerbation, 13 had rhinovirus infection and 1 had influenza virus infection according to multiplex PCR analysis of nasopharyngeal swab samples. The spirometric indices were lower in children with asthma exacerbation than in those with stable asthma and controls.

**TABLE 1:** Subjects' characteristics (*N* = 95)

	Asthma exacerbation (*N* = 22)	Stable asthma (*N* = 67)	Control (*N* = 6)
Age, years	9.0 (6.4/10.9)^*^	8.0 (6.6/9.7)^*^	13.2 (10.7/14.9)
Male sex, *n* (%)	15 (68.2)	50 (74.6)	4 (66.7)
Total IgE, IU/ml	484 (230/973)	439 (201/919)	448 (110/1065)
Blood eosinophil	460 (208/663)	420 (290/710)	220 (180/430)
Sputum eosinophil, %	3.0 (0.0/21.5)	2.0 (0.0/10.0)	2.0 (0.0/4.8)
Infected pathogen			
Rhinovirus	13 (59.1)		
Influenza	1 (4.5)		
Pulmonary function			
FEV_1_, % predicted	72.6 ± 21.4^*,**^	95.6 ± 15.9	110.4 ± 8.4
Δ FEV_1_, %	4.6 (2.0/19.6)	7.5 (4.0/13.3)^*^	1.6 (−0.2/3.6)
FEV_1_/FVC	77.4 (65.7/82.8)^*,**^	81.4 (73.5/85.4)	90.3 (86.8/92.2)

*Note*: All subjects are atopic.

Abbreviations: FEV_1_, forced expiratory volume in one second; FVC, forced vital capacity.

^*^ *p* < 0.05 versus healthy control.

^**^ *p* < 0.05 versus stable asthma.

### 3.2 Taxon distribution and α, β‐diversity

The taxa composition was visualised as stacked bar graphs at the phylum and genus levels, as shown in Figures S1 and S2. At the phylum level, *Proteobacteria* was more abundant and *Saccharibacteria_TM7* and *Actinobacteria* were less abundant in children with asthma exacerbation. At the genus level, *Veillonella*, *Neisseria*, *Haemophilus*, *Fusobacterium*, *Oribacterium*, *Campylobacter*, and *Capnocytophaga* showed higher abundance and *Saccharimonas*, *Rothia*, *Porphyromonas*, *Gemella*, and *Actinomyces* showed lower abundance in this group.

The α‐diversity indices about species richness including ACE, Chao 1, and Jackknife and species diversity including NPShannon, Shannon, Simpson, and phylogenetic diversity did not differ between children with asthma exacerbation and those with stable asthma (Table S1). The β‐diversity indices including Jensen‐Shannon, Bray‐Curtis, Generalised UniFrac, and UniFrac significantly differed between children with asthma exacerbation and those with stable asthma (Table S2).

### 3.3 Microbial candidates in asthma exacerbation

As the β‐diversity indices between children with asthma exacerbation and those with stable asthma were significantly different, we identified the prominently increased or decreased the microbial candidates in children with asthma exacerbation compared to in those with stable asthma by LEfSe and SIMPER analysis.

**FIGURE 1:**
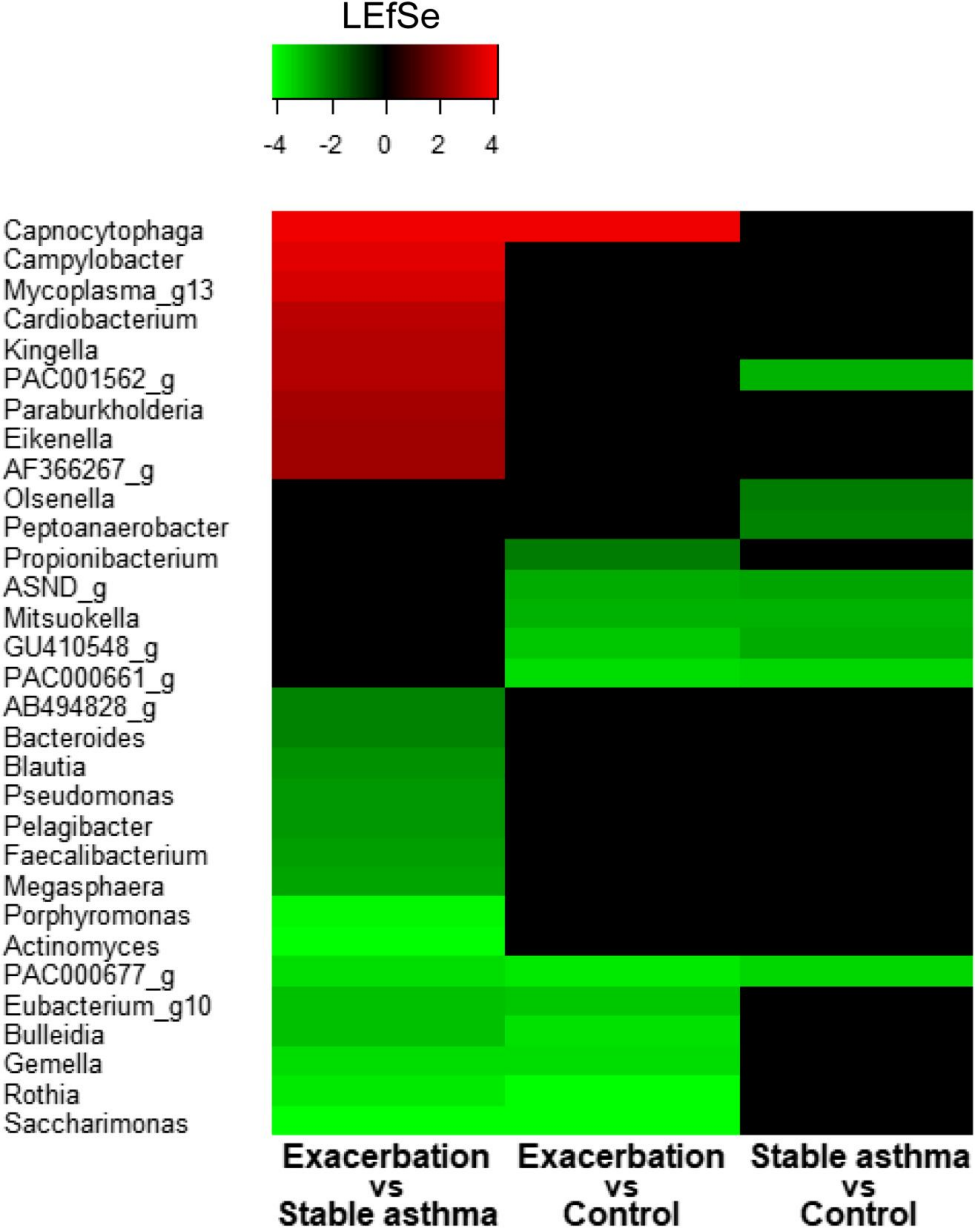
Heatmap plotted from linear discriminant analysis effect size (LEfSe) analysis at the genus level between groups. *Capnocytophaga* was increased in children with asthma exacerbation compared to in those with stable asthma and controls, whereas *Saccharominas*, *Rothia*, *Gemella*, *Bulleidia*, and *Eubacterium_g10* were decreased

LEfSe analysis showed that *Capnocytophaga* was the only prominently increased the microbial candidate in children with asthma exacerbation compared to in those with stable asthma and controls, whereas the prominently decreased the microbial candidates in those with asthma exacerbation were *Saccharimonas*, *Rothia*, *Gemella*, *Bulleidia*, and *Eubacterium_g10* (Figure 1).

**FIGURE 2:**
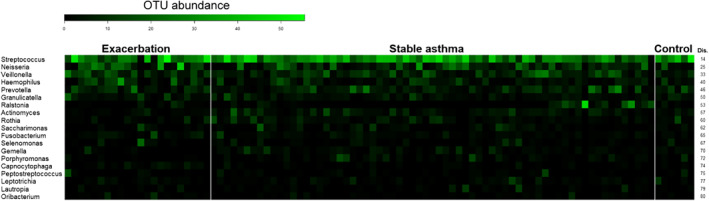
Relative abundance (operational taxonomic unit abundance) of discriminant microbiota plotted from SIMPER analysis at the genus level between groups. The 19 microbial candidates listed in the heatmap explained the difference between asthma exacerbation and stable asthma with up to 80% cumulative dissimilarity (Dis.)

According to SIMPER analysis, the microbial candidates that could explain the 80% dissimilarity in microbiome composition between those with asthma exacerbation and those with stable asthma were *Streptococcus*, *Neisseria*, *Veillonella*, *Haemophilus*, *Prevotella*, *Granulicatella*, *Ralstonia*, *Actinomyces*, *Rothia*, *Saccharimonas*, *Fusobacterium*, *Selemonas*, *Gemella*, *Porphyromonas*, *Capnocytophaga*, *Peptostreptococcus*, *Leptotrichia*, *Lautrophia*, and *Oribacterium* (Figure 2).

**FIGURE 3:**
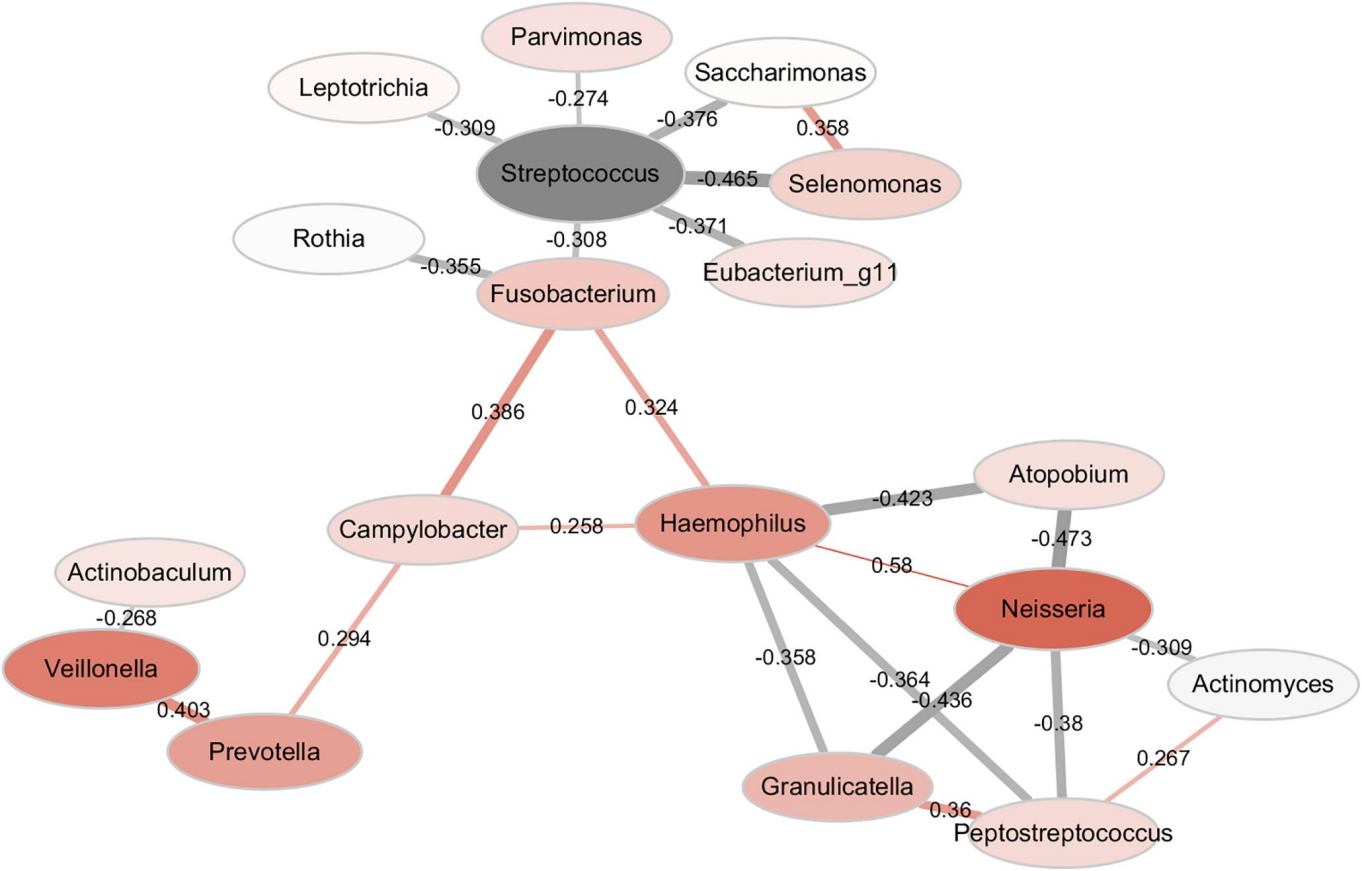
Network analysis of microbiota in asthma exacerbation. Node size is proportional to the mean relative abundance. Node colour (red represents increased microbiota and grey represents decreased microbiota in asthma exacerbation) and node hue is proportional to the difference in microbiota relative abundance between asthma exacerbation and stable asthma. Each edge: a significant correlation coloured to indicate either positivity (red) or negativity (grey). Edge width and transparency are proportional to the absolute value of the correlation coefficient. Correlations were determined with SparCC with a correlation cut‐off *R* value of greater than 0.25 or less than −0.25

To clarify the association among the microbes during acute exacerbation, a microbiota network was generated in children with asthma exacerbation (Figure 3). Significantly correlated the microbial candidates included *Campylobacter*, *Haemophilus*, *Neisseria*, *Granulicatella*, *Peptostreptococcus*, *Fusobacterium*, and *Streptococcus*, among others.

Finally, we selected the 26 microbial candidates significantly increased, decreased, or correlated with each other during acute exacerbation of asthma in children through LEfSE, SIMPER, and network analysis.

### 3.4 Relationship between microbial candidates and significant cytokines

**FIGURE 4:**
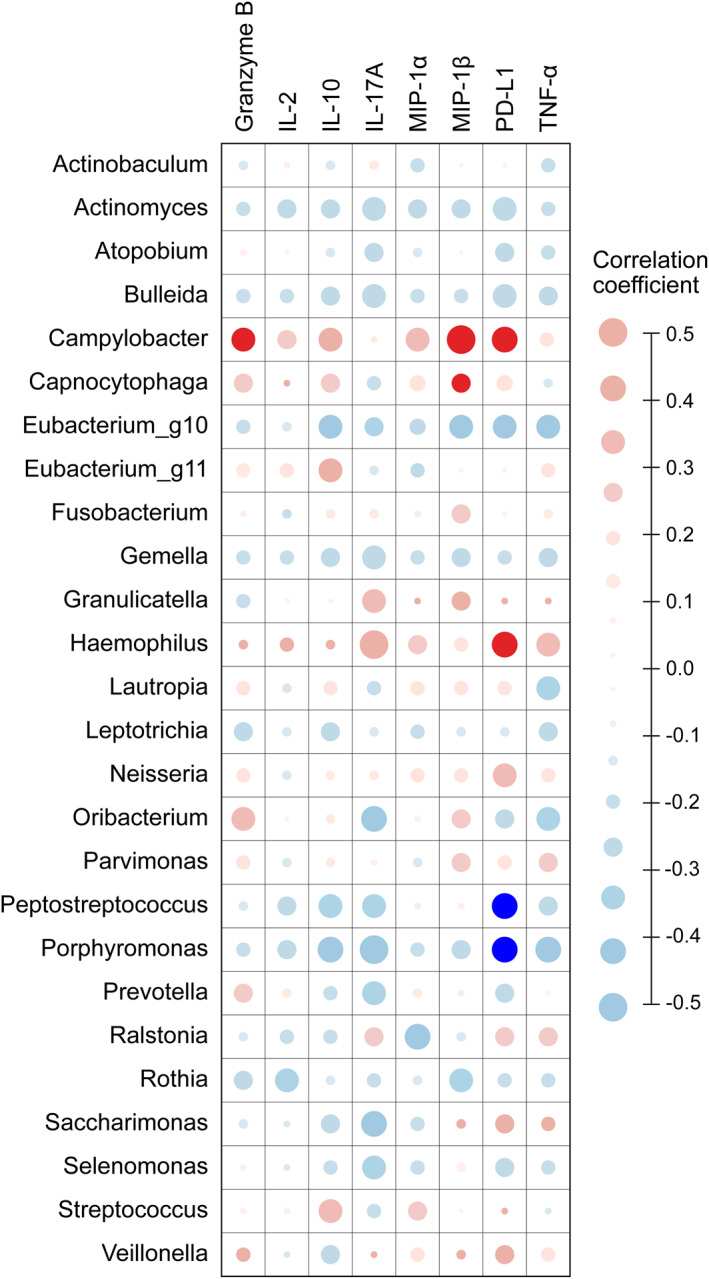
Correlation between microbial candidates distinct in asthma exacerbation from LEfSe, SIMPER, and network analysis using SparCC and prominently increased inflammatory cytokines in asthma exacerbation. Node size is proportional to Spearman's rank correlation coefficient. Red bar: positive correlation; blue bar: negative correlation; dark coloured node: false discovery rate (FDR) *p* < 0.05; light coloured node: FDR *p* ≥ 0.05

The significantly increased inflammatory cytokines in children with asthma exacerbation compared as those with stable asthma were granzyme B, IL‐2, IL‐10, IL‐17A, MIP‐1α, MIP‐1β, PD‐L1, and tumour necrosis factor‐α, as shown in Table S3. Because the airway microbiome and inflammatory cytokines in asthma have been shown to be closely related,^20^ we analysed the correlation between these inflammatory cytokines and the 26 microbial candidates in children with asthma shown, as shown in Figure 4. *Campylobacter* was positively correlated with granzyme B, MIP‐1β, and PD‐L1. *Capnocytophaga* was positively correlated with MIP‐1β, and *Haemophilus* was positively correlated with PD‐L1. *Peptostreptococcus* and *Porphyromonas* were negatively correlated with PD‐L1.

As MIP‐1β has been known to play a role in eosinophilic recruitment,^21^ we analysed the correlation among levels of MIP‐1β and sputum eosinophil counts (Spearman coefficient *r* = 0.478, *p* = 0.028) in children with asthma exacerbation. *Campylobacter* and *Capnocytophaga*, which were significantly correlated with MIP‐1β in this study, showed similar results in correlation analysis with sputum eosinophil counts; only *Campylobacter* showed a significant correlation with sputum eosinophils (Spearman coefficient *r* = 0.462, *p* = 0.035).

### 3.5 Prediction of metagenome function in asthma exacerbation

**FIGURE 5:**
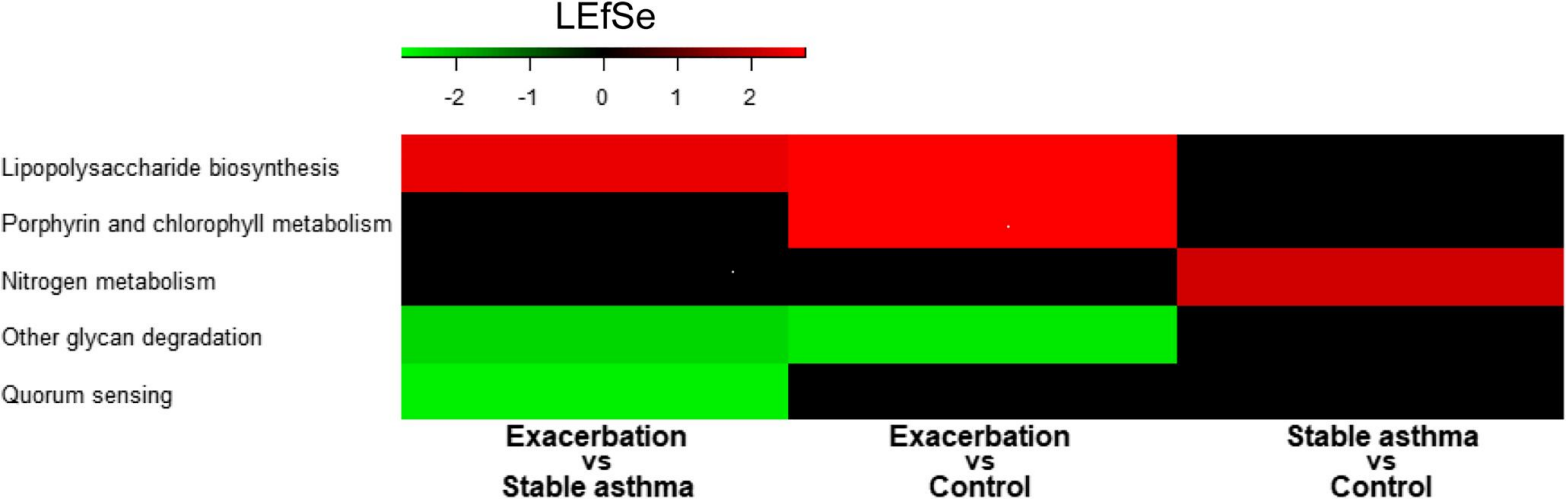
Heatmap plotted from linear discriminant analysis effect size analysis of predicted functional profiles using PICRUSt between groups. Lipopolysaccharide biosynthesis was increased and glycan degradation was decreased in children with asthma exacerbation compared to in children with stable asthma and controls

The predicted function of the airway microbiome was assessed to determine whether it was differed between children with asthma exacerbation and children with stable asthma and controls using PICRUSt, as shown in Figure 5. Lipopolysaccharide (LPS) biosynthesis was increased, whereas glycan degradation was decreased in children with asthma exacerbation compared to in those with stable asthma and control.

## 4 DISCUSSION

During acute exacerbation of allergic asthma in children, gram‐negative microbes were increased prominently, showing a relationship with increased granzyme B, MIP‐1β, and PD‐L1 in induced sputum. Among the increased gram‐negative microbes, *Campylobacter* was associated with increased MIP‐1β and the sputum eosinophils, indicating that it plays a role in asthma exacerbation in children. Predictive metagenome functional analysis showed increased LPS biosynthesis and decreased glycan degradation in children with asthma exacerbation.

*Campylobacter* was increased significantly according to LEfSe analysis and showed a significant correlation with several inflammatory cytokines including granzyme B, MIP‐1β, and PD‐L1, which were increased in asthma exacerbation. Although *Campylobacter* is typically considered as a gastrointestinal pathogen, it can be increased in lung in chronic obstructive pulmonary disease and interstitial lung disease.^22^, ^23^ This finding can be explained by the fact that gastroesophageal reflux or oral aspiration may contribute to exacerbation of chronic lung disease.^24^ In the gut–lung axis, gut microbes may influence the lungs via the spilling over of increased gut microbes and their inflammatory mediators in the body.^25^ Although *Campylobacter* has not been widely studied in asthma, it may play a role in asthma exacerbation as a driving microbe, as it showed a close correlation with other microbes in bacterial network analysis.

MIP‐1β was recently reported to recruit eosinophils into the airway and was found to be increased in asthma exacerbation in an eosinophil‐dominant biological cluster.^21^, ^26^ *Campylobacter* can induce eosinophil chemotaxis as well as degranulation and release of eosinophil cationic proteins and major basic proteins.^27^ Since *Campylobacter* was correlated with both MIP‐1β and eosinophils in our study; we hypothesised that MIP‐1β mediated the relationship between *Campylobacter* and eosinophils. Eosinophil inflammation is exaggerated upon exposure to allergens, and allergic asthma showing allergen sensitisation is more important in children compared to other asthma phenotypes including neutrophil‐ or obesity‐related asthma.^28^ Therefore, the identification of *Campylobacter* provides a new perspective in children with allergic asthma exacerbation.

Granzyme B signalling was reported to be induced by *Campylobacter* infection in humans, which may be related to the inflammatory response.^29^ Granzyme B was suggested as a novel target molecule in allergic pulmonary inflammation^30^ and was reported to be increased in fatal asthma, as it was delivered into target cells to exert its cytotoxic function and it cleaved extracellular matrix components, contributing to remodelling in chronic inflammation.^31^ Considering these reports, the relationship between increased granzyme B and *Campylobacter* in our subjects may play a role during asthma exacerbation.

PD‐L1 was shown to be correlated with most of the microbial candidates in our study. PD‐L1 has been reported to be an important target molecule in cancer therapy, and the gut microbiome can affect anti‐PD‐L1 treatment.^32^ Although few studies have evaluated the relationship between PD‐L1 and asthma, PD‐L1 and PD‐L2 are known to affect asthma differently and PD‐L1 may strengthen Th2 inflammation and increase airway hyper‐responsiveness in asthma.^33^ From the perspective of acute infection, PD‐L1 was reported to suppress CD8 T‐cell immunity, preventing the clearance of infected pathogens.^34^, ^35^ The dual roles of PD‐L1 in asthma, which are strengthening Th2 inflammation and weakening innate immunity from infected pathogens, can explain its contribution to asthma exacerbation.

*Haemophilus* was increased in children with asthma exacerbation compared to in those with stable asthma according to SIMPER analysis with 7% dissimilarity and showed a significant correlation with PD‐L1. This increase in *Haemophilus* is consistent with the findings of previous studies of severe asthma, which showed that *Haemophilus* suppressed host innate immunity and may contribute to the persistence of infection in allergic asthma.^36^, ^37^, ^38^ *Haemophilus* was shown to be significantly correlated with other microbiotas during asthma exacerbation in bacterial network analysis, which was also found in another study of neutrophilic asthma. The most common causative factor of asthmas exacerbation is respiratory infection such as with *mycoplasma* and rhinovirus, for which the infective environment can be neutrophil dominant.^36^

In contrast, *Haemophilus* species levels differ in infants and children with asthma according to age and are related to a reduced risk of exacerbation in children.^6^ This discrepancy may have resulted from the use of different sampling time points. We collected samples during asthma exacerbation, whereas others studies performed regular sampling independently of asthma exacerbation. In addition, we collected induced sputum, whereas other studies used nasal swabbed samples, which may be resulted in differences between the results. Therefore, our data more accurately explains the pathophysiology of asthma exacerbation, whereas other studies predicted the development of asthma exacerbation.^7^

*Capnocytophaga* and *Porphyromonas* showed a prominent change and were the microbial candidates involved in asthma exacerbation according to LEfSe and SIMPER analysis. However, these genera were present in low abundance and not related in the bacterial network. Therefore, their influence might be smaller than those of other candidate microbes, which is supported by previous studies. However, further studies of these microbes may improve the understanding of the mechanism of asthma exacerbation. The microbes were found in the upper airway or oral cavity, where respiratory infection related to asthma exacerbation can occur.^39^, ^40^

Although microbial functional analysis is helpful for understanding the interactions between microorganisms and host health, we did not perform metagenomic functional analysis because of its high cost and requirement for large amounts of DNA.^41^ Therefore, we used the PICRUSt software program, which is designed to predict the metagenome functional content from marker genes using 16S rRNA surveys.^17^ LPS was found to be increased, possibly because of increased levels of gram‐negative microbes during asthma exacerbation. A recent study reported that bacterial LPS binding enhanced virus stability and promoted viral infectivity in the gastrointestinal tract.^42^ This finding should be further evaluated in the respiratory tract because respiratory viral infection is an important risk factor of asthma exacerbation. In our study, glycan degradation was decreased, reducing short‐chain fatty acids reduced. Short‐chain fatty acids are known to promote regulatory T lymphocytes, which can protect against asthma development.^43^, ^44^

There were some limitations to this study. First, we could not control systemic steroid uses for asthma exacerbation, which may have influenced the airway microbial composition. However, this limitation is inevitable in clinical research of asthma exacerbation. To minimise this effect, we collected induced sputum within 24 h of hospital visits. In addition, we could not evaluate the airway microbiome according to age. Considered that there was no difference in age between children with asthma exacerbation and those with stable asthma and that most children were more than 8 years old, an age at which the airway microbiome changes, this confounding effect may have been small in this study.^5^

This is the first study to evaluate the airway microbiome using induced sputum, which can reflect the lower airway state in children with asthma. Sputum induction was performed at the beginning of asthma exacerbation, enabling accurate assessment of the airway inflammatory status in children with asthma exacerbation. As we identified important the microbial candidates through a multifaceted analysis including LEfSe, SIMPER, and bacterial network analysis, and identified cytokines related to these candidates by multiple correction, our results provide precise information for understating asthma exacerbation relating and its relationship with the airway microbiome in children.

**FIGURE 6:**
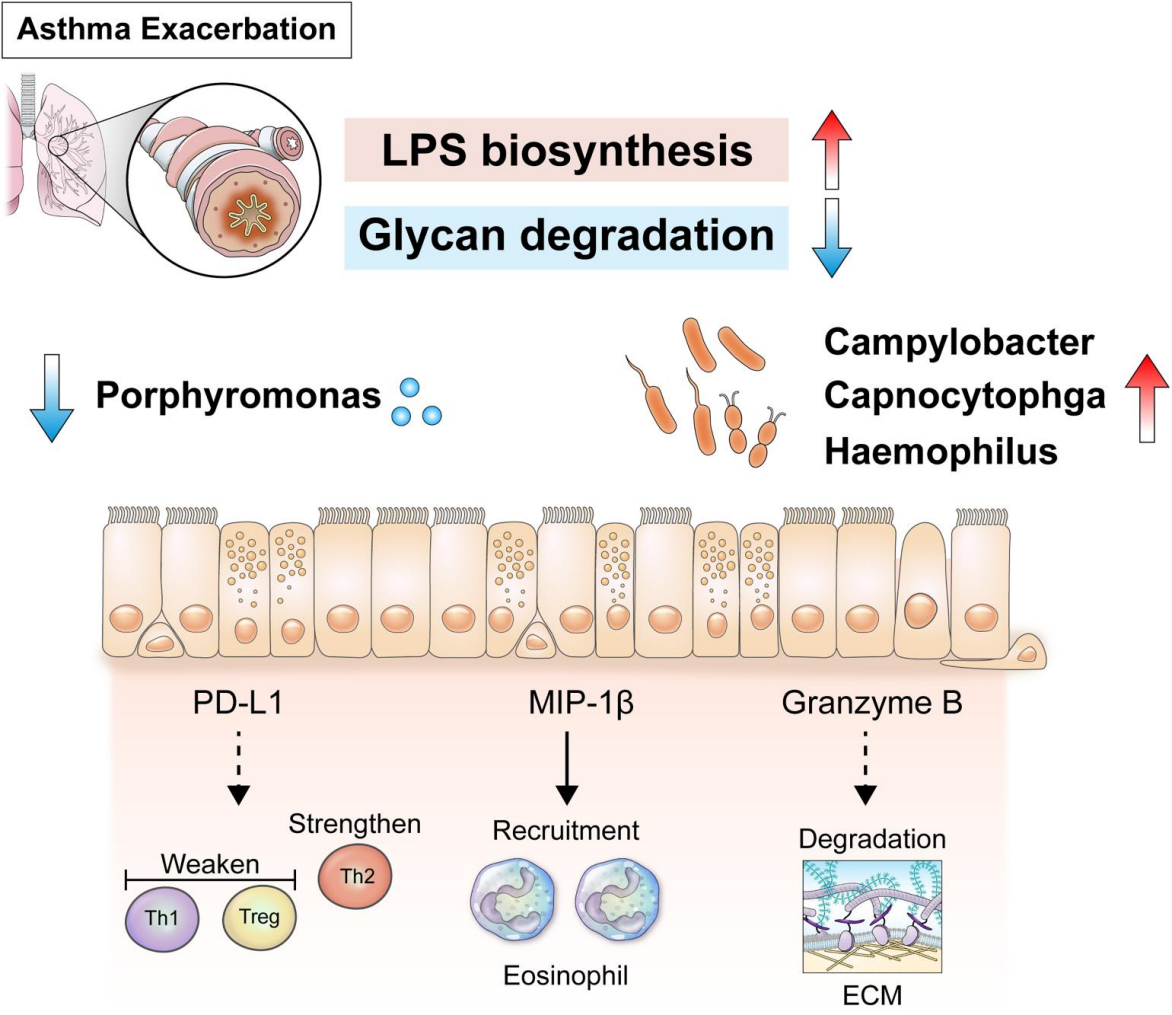
Gram‐negative microbes in the lower airway that increase lipopolysaccharide biosynthesis and decrease glycan degradation may promote acute exacerbation of allergic asthma in children via inflammatory cytokines including programmed death‐ligand 1, macrophage inflammatory proteins (MIP)‐1β, and granzyme B. Among the increased gram‐negative microbes, *Campylobacter* was associated with increased MIP‐1β and sputum eosinophils, indicating that this genus plays a role in asthma exacerbation in children

In conclusion, gram‐negative microbes in the lower airway were related to the acute exacerbation state in children with asthma by increasing inflammatory cytokines such as granzyme B, MIP‐1β, and PD‐L1 and changing the metabolic status such as LPS biosynthesis and glycan degradation (Figure 6). These microbes and related cytokines and functional pathways may play a role in asthma exacerbation in children.

## CONFLICT OF INTEREST

All authors of this study declare that there is no conflict of interest.

## Supporting information

Supporting Information S1

Figure S1

Figure S2

Table S1

Table S2

Table S3
