# Supplementary material for: Gram‐negative microbiota is related to acute exacerbation in children with asthma
Source: Clin Transl Allergy. 2021 Oct 12;11(8):e12069. doi: 10.1002/clt2.12069 (PMC8507365; doi:10.1002/clt2.12069)
Supplement: Supplementary file 1 — Supporting Information S1 [file CLT2-11-e12069-s003.doc]

**Supporting Methods**

**Sputum induction and processing**

All children were instructed to wash their mouths thoroughly with water, after which they inhaled a 3% saline solution nebulized in an ultrasonic nebulizer (NE-U12; Omron Co., Tokyo, Japan) at maximum output at room temperature. The children were encouraged to cough deeply at 3-min intervals thereafter. Sputum samples were kept at 4°C for no more than 2 h before further processing. A portion of the samples was diluted with a phosphate-buffered saline (PBS) solution containing 10 mmol/L of dithiothreitol (WAKO Pure Chemical Industries Ltd, Osaka, Japan) for cell count and microbiome analysis. For analysis of cytokines, another portion of the samples was gently vortexed at room temperature for 20 min after dilution with PBS solution containing 10 mmol/L of dithiothreitol. Sputum aliquots for microbiome and cytokine analysis were stored at -70°C.

**DNA extraction, PCR amplification and sequencing**

For microbiome analysis, total DNA was extracted from the portion of the sputum sample using the FastDNA® SPIN Kit for Soil (MP Biomedicals, USA), in accordance with the manufacturer’s instruction. PCR amplification was performed using fusion primers targeting from V3 to V4 regions of the 16S rRNA gene with the extracted DNA. For bacterial amplification, fusion primers of 341F (5’-AATGATACGGCGACCACCGAGATCTACAC-XXXXXXXX-TCGTCGGCAGCGTC-AGATGTGTATAAGAGACAG-CCTACGGGNGGCWGCAG-3’; underlining sequence indicates the target region primer) and 805R (5’- CAAGCAGAAGACGGCATACGAGAT-XXXXXXXX-GTCTCGTGGGCTCGG-AGATGTGTATAAGAGACAG-GACTACHVGGGTATCTAATCC-3’). The Fusion primers are constructed in the following order which is P5 (P7) graft binding, i5 (i7) index, Nextera consensus, Sequencing adaptor, and Target region sequence.

The amplifications were carried out under the following conditions: initial denaturation at 95 °C for 3min, followed by 25 cycles of denaturation at 95 °C for 30 s, primer annealing at 55 °C for 30 s, and extension at 72 °C for 30 s, with a final elongation at 72 °C for 5 min.

The PCR product was confirmed by using 1% agarose gel electrophoresis and visualized under a Gel Doc system (BioRad, Hercules, CA, USA). The amplified products were purified with the Clean PCR (CleanNA). Equal concentrations of purified products were pooled together and removed short fragments (non-target products) with Clean PCR (CleanNA). The quality and product size were assessed on a Bioanalyzer 2100 (Agilent, Palo Alto, CA, USA) using a DNA 7500 chip. Mixed amplicons were pooled and the sequencing was carried out at Chunlab, Inc. (Seoul, Korea), with Illumina MiSeq Sequencing system (Illumina, USA) according to the manufacturer’s instructions.

**Microbiome data analysis**

Processing raw reads started with quality check and filtering of low quality (<Q25) reads by Trimmomatic ver. 0.32. [1] After quality check pass, paired-end sequence data were merged together using fastq_mergepairs command of VSEARCH version 2.13.4 [2] with default parameters. Primers were then trimmed with the alignment algorithm of Myers & Miller [3] at a similarity cut off of 0.8. Non-specific amplicons that do not encode 16S rRNA were detected by nhmmer [4] in HMMER software package ver. 3.2.1 with hmm profiles. Unique reads were extracted and redundant reads were clustered with the unique reads by derep_full length command of VSEARCH. [2] The EzBioCloud 16S rRNA database [5] was used for taxonomic assignment using usearch_global command of VSEARCH [2] followed by more precise pairwise alignment. [3] Chimeric reads were filtered on reads with <97% similarity by reference based chimeric detection using UCHIME algorithm [6] and the non-chimeric 16S rRNA database from EzBioCloud. After chimeric filtering, reads that are not identified to the species level (with <97% similarity) in the EzBioCloud database were compiled and cluster_fast command [2] was used to perform de-novo clustering to generate additional OTUs. Finally, OTUs with single reads (singletons) are omitted from further analysis.

The secondary analysis which includes diversity calculation and biomarker discovery was conducted by in-house programs of Chunlab, Inc (Seoul, South Korea). The alpha diversity indices (ACE [7], Chao1 [8], Jackknife [9], Shannon [10], NPShannon [11], Simpson [12] and Phylogenetic diversity [13]), rarefaction curves [14], rank abundance curves [15] were estimated. To visualize the sample differences, beta diversity distances were calculated by several algorithms (Jensen-Shannon [17], Bray-Curtis [18], Generalized UniFrac [19], Fast UniFrac [20]).

**Supporting References**

1. Bolger AM, Lohse M, Usadel B. Trimmomatic: a flexible trimmer for Illumina sequence data. *Bioinformatics*. 2014;30(15):2114-2120.

2. Rognes T, Flouri T, Nichols B, Quince C, Mahé F. VSEARCH: a versatile open source tool for metagenomics. *PeerJ*. 2016;4:e2584.

3. Myers EW, Miller W. Optimal alignments in linear space. *Bioinformatics*. 1988;4(1):11-17.

4. Wheeler TJ, Eddy SR. nhmmer: DNA homology search with profile HMMs. *Bioinformatics*. 2013;29(19):2487-2489.

5. Yoon SH, Ha SM, Kwon S, et al. Introducing EzBioCloud: a taxonomically united database of 16S rRNA gene sequences and whole-genome assemblies. *Int J Syst Evol Microbiol*. 2017;67(5):1613-1617.

6. Edgar RC, Haas BJ, Clemente JC, Quince C, Knight R. UCHIME improves sensitivity and speed of chimera detection. *Bioinformatics*. 2011;27(16):2194-2200.

7. Chao A, Lee S-M. Estimating the number of classes via sample coverage. *Journal of the American statistical Association*. 1992;87(417):210-217.

8. Chao A. Estimating the population size for capture-recapture data with unequal catchability. *Biometrics*. 1987;43(4):783-791.

9. Burnham KP, Overton WS. Robust estimation of population size when capture probabilities vary among animals. *Ecology*. 1979;60(5):927-936.

10. Magurran AE. *Measuring biological diversity.* John Wiley & Sons; 2013.

11. Chao A, Shen T-J. Nonparametric estimation of Shannon’s index of diversity when there are unseen species in sample. *Environmental and ecological statistics*. 2003;10(4):429-443.

12. Faith DP. Conservation evaluation and phylogenetic diversity. *Biological conservation*. 1992;61(1):1-10.

13. Heck Jr KL, van Belle G, Simberloff D. Explicit calculation of the rarefaction diversity measurement and the determination of sufficient sample size. *Ecology*. 1975;56(6):1459-1461.

14. Whittaker RH. Dominance and diversity in land plant communities: numerical relations of species express the importance of competition in community function and evolution. *Science*. 1965;147(3655):250-260.

15. Lin J. Divergence measures based on the Shannon entropy. *IEEE Transactions on Information theory*. 1991;37(1):145-151.

16. Beals EW. Bray-Curtis ordination: an effective strategy for analysis of multivariate ecological data. *Advances in ecological research.* Vol 14: Elsevier; 1984:1-55.

17. Chen J, Bittinger K, Charlson ES, et al. Associating microbiome composition with environmental covariates using generalized UniFrac distances. *Bioinformatics*. 2012;28(16):2106-2113.

18. Hamady M, Lozupone C, Knight R. Fast UniFrac: facilitating high-throughput phylogenetic analyses of microbial communities including analysis of pyrosequencing and PhyloChip data. *The ISME journal*. 2010;4(1):17-27.

**Supporting Figure Legends**

**Supporting Figure 1.** Stacked bars showing composition of microbiota in phylum levels among the groups including asthma exacerbation, stable asthma and control.

**Supporting Figure 2.** Stacked bars showing composition of microbiota in genus levels among the groups including asthma exacerbation, stable asthma and control.
