# Supplementary figures and images for: Gram‐negative microbiota is related to acute exacerbation in children with asthma
Source: Clin Transl Allergy. 2021 Oct 12;11(8):e12069. doi: 10.1002/clt2.12069 (PMC8507365; doi:10.1002/clt2.12069)

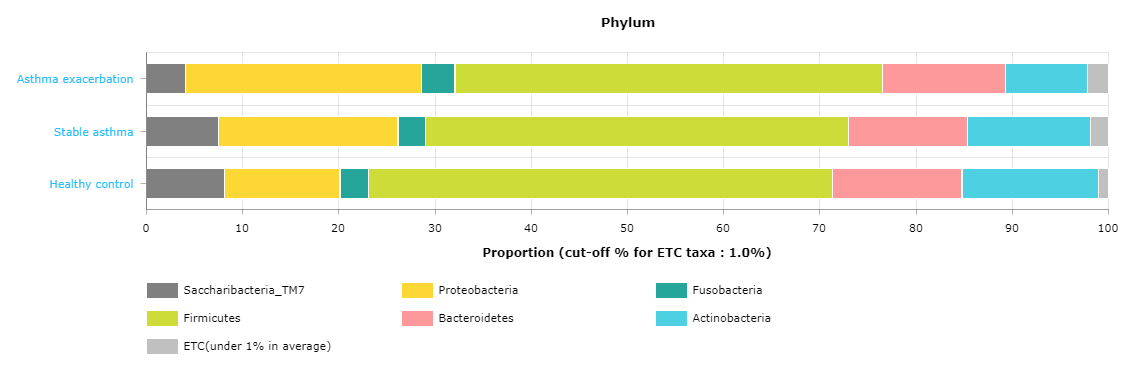

Supplement: Supplementary file 2 — Figure S1 [file CLT2-11-e12069-s002.tiff]

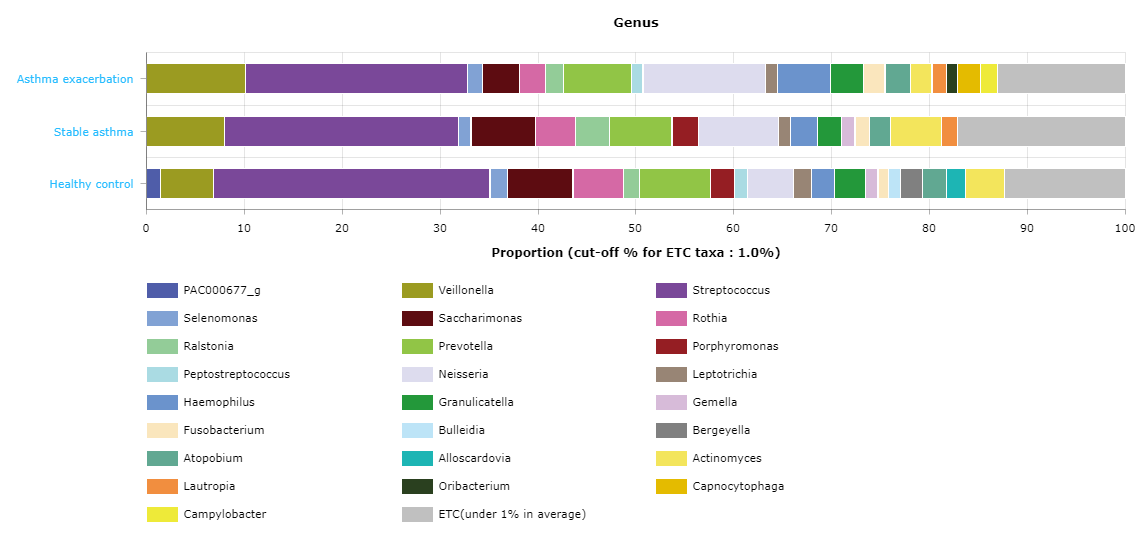

Supplement: Supplementary file 3 — Figure S2 [file CLT2-11-e12069-s006.tiff]
