# Supplementary material for: Gram‐negative microbiota is related to acute exacerbation in children with asthma
Source: Clin Transl Allergy. 2021 Oct 12;11(8):e12069. doi: 10.1002/clt2.12069 (PMC8507365; doi:10.1002/clt2.12069)
Supplement: Supplementary file 4 — Table S1 [file CLT2-11-e12069-s001.docx]

| **Supporting Table 1** Alpha diversity of airway microbiome among the groups | | | | |
| --- | --- | --- | --- | --- |
|  |  | Asthma exacerbation  (N = 22) | Stable asthma  (N = 67) | Control  (N = 6) |
| Species richness | ACE | 284.6 (245.6/352.6) | 290.4 (246.6/375.0) | 352.5 (269.5/469.6) |
|  | Chao 1 | 276.9 (239.7/339.2) | 281.4 (238.6/362.5) | 344.9 (236.2/448.3) |
|  | Jackknife | 295.0 (253.0/365.5) | 301.0 (257.0/387.0) | 363.0 (280.0/492.0) |
|  | Number of OUTs | 269.0 (232.5/333.0) | 277.0 (230.0/355.0) | 340.0 (257.0/436.0) |
| Diversity index | NPShannon | 3.53 (3.27/3.76) | 3.39 (2.93/3.71) | 3.64 (3.47/3.88) |
|  | Shannon | 3.51 (3.27/3.75) | 3.38 (2.92/3.69) | 3.62 (3.46/3.87) |
|  | Simpson | 0.06 (0.05/0.09) | 0.07 (0.06/0.1) | 0.06 (0.05/0.09) |
|  | Phylogenetic diversity | 428.5 (360.0/505.0) | 448.0 (371.0/512.0) | 445.5 (412.0/572.0) |
| ACE, Abundance-based coverage estimators; OUT, Operational taxonomic unit.  Wilcoxon rank-sum test was done among 2 groups. | | | | |
