# Supplementary material for: Gram‐negative microbiota is related to acute exacerbation in children with asthma
Source: Clin Transl Allergy. 2021 Oct 12;11(8):e12069. doi: 10.1002/clt2.12069 (PMC8507365; doi:10.1002/clt2.12069)
Supplement: Supplementary file 5 — Table S2 [file CLT2-11-e12069-s005.docx]

| **Supporting Table 2** PERMANOVA analysis with beta diversity of airway microbiome among the groups | | | |
| --- | --- | --- | --- |
|  | Asthma exacerbation  vs.  Stable asthma | Asthma exacerbation  vs.  Control | Stable asthma  vs.  Control |
| Jensen-Shannon | 0.001* | 0.050 | 0.822 |
| Bray-Curtis | 0.005* | 0.274 | 0.866 |
| Generalized UniFrac | 0.001* | 0.093 | 0.704 |
| UniFrac | 0.031* | 0.198 | 0.859 |
| * p < 0.01 from PERMANOVA analysis among the groups. | | | |
