# Supplementary material for: Gram‐negative microbiota is related to acute exacerbation in children with asthma
Source: Clin Transl Allergy. 2021 Oct 12;11(8):e12069. doi: 10.1002/clt2.12069 (PMC8507365; doi:10.1002/clt2.12069)
Supplement: Supplementary file 6 — Table S3 [file CLT2-11-e12069-s004.docx]

| **Supporting Table 3** Inflammatory cytokines between asthma exacerbation and stable asthma | | | | |
| --- | --- | --- | --- | --- |
|  | Asthma exacerbation (N = 21) | Stable asthma (N = 63) | *p* | FDR *p*^*^ |
| CD40 | 1178.9 (386.3/2261.9) | 509.3 (233.6/1272.1) | 0.143 | 0.215 |
| GM-CSF | 24.9 (7.5/155.7) | 11.4 (2.7/32.9) | 0.062 | 0.114 |
| Granzyme B^*^ | 228.4 (36.4/618.3) | 26.2 (11.7/82.4) | < 0.001 | < 0.001 |
| INF-α | 5.7 (3.5/8.1) | 2.8 (1.1/5.6) | 0.041 | 0.098 |
| IFN-γ | 6.5 (3.1/16.3) | 6.3 (1.9/9.6) | 0.323 | 0.431 |
| IL-1α | 100.9 (45.9/181.2) | 81.5 (43.9/157.5) | 0.451 | 0.515 |
| IL-1β | 41.9 (30.5/132.7) | 40.1 (18.8/112.9) | 0.480 | 0.524 |
| IL-1 Ra | 42474.2 ± 29055.0 | 41299.5 ± 18478.3 | 0.910 | 0.910 |
| IL-2^*^ | 15.6 (4.5/37.4) | 4.6 (1.4/9.8) | 0.005 | 0.020 |
| IL-4 | 1.7 (0.5/3.8) | 0.9 (0.4/1.8) | 0.199 | 0.281 |
| IL-6 | 50.1 (6.4/369.5) | 25.4 (8.7/50.0) | 0.073 | 0.125 |
| IL-8 | 1228.7 (132.9/5126.9) | 216.3 (102.5/663.3) | 0.027 | 0.072 |
| IL-10^*^ | 47.8 (11.7/168.8) | 5.3 (4.6/39.1) | 0.008 | 0.024 |
| IL-12 p70 | 6.4 (4.9/21.6) | 6.9 (2.8/11.1) | 0.595 | 0.621 |
| IL-13 | 50.1 ± 27.4 | 41.4 ± 21.8 | 0.054 | 0.114 |
| IL-15 | 2.9 (1.1/6.7) | 1.9 (0.4/3.1) | 0.135 | 0.215 |
| IL-17A^*^ | 4.6 (3.7/9.0) | 1.2 (0.9/3.1) | 0.003 | 0.014 |
| IL-33 | 31.1 (14.2/52.8) | 19.6 (7.3/40.7) | 0.37 | 0.467 |
| CXCL10 | 33.5 (7.7/499.1) | 11.9 (5.1/72.8) | 0.062 | 0.114 |
| MCP-1 | 15.5 (10.9/24.9) | 12.7 (7.4/69.1) | 0.435 | 0.515 |
| MIP-1α^*^ | 26.1 (18.9/62.4) | 19.3 (12.4/27.4) | 0.008 | 0.024 |
| MIP-1β^*^ | 929.5 (455.1/1684.3) | 282.2 (214.8/418.4) | < 0.001 | < 0.001 |
| PD-L1^*^ | 237.2 (69.6/430.9) | 69.4 (17.5/108.8) | 0.002 | 0.014 |
| TNF- α^*^ | 14.4 (5.9/20.8) | 2.7 (2.1/7.6) | 0.003 | 0.014 |
| CD, Cluster of differentiation; GM-CSF, Granulocyte-macrophage colony-stimulating factor; IFN, Interferon; IL, Interleukin; CXCL, C-X-C motif chemokine; MCP, Monocyte chemoattractant protein; MIP, Macrophage Inflammatory Proteins; PD-L, Programmed death-ligand; TNF, Tumor necrosis factor.  ^*^FDR *p* (significant in < 0.05) is a false discovery rate correction of original *p*. | | | | |
